# Gefitinib provides similar effectiveness and improved safety than erlotinib for east Asian populations with advanced non–small cell lung cancer: a meta-analysis

**DOI:** 10.1186/s12885-018-4685-y

**Published:** 2018-08-02

**Authors:** Wenxiong Zhang, Yiping Wei, Dongliang Yu, Jianjun Xu, Jinhua Peng

**Affiliations:** Department of thoracic surgery, The second affiliated hospital of Nanchang University, 1 Min De Road, Nanchang, 330006 China

**Keywords:** Gefitinib, Erlotinib, Non-small cell lung cancer, East Asian populations, Targeted therapy, Meta-analysis

## Abstract

**Background:**

The first-generation epidermal growth factor receptor tyrosine kinase inhibitors gefitinib and erlotinib have both been proven effective for treating advanced non–small cell lung cancer (NSCLC), especially in East Asian patients. We conducted this meta-analysis to compare their efficacy and safety in treating advanced NSCLC in this population.

**Methods:**

We systematically searched PubMed, ScienceDirect, The Cochrane Library, Scopus, Ovid MEDLINE, Embase, Web of Science, and Google Scholar for the relevant studies. Overall survival (OS), progression-free survival (PFS), objective response rate (ORR), disease control rate (DCR), and adverse effects (AEs) were analyzed as primary endpoints.

**Results:**

We identified 5829 articles, among which 31 were included in the final analysis. Both gefitinib and erlotinib were effective for treating advanced NSCLC, with comparable PFS (95% confidence interval [CI]: 0.97–1.10, *p* = 0.26), OS (95% CI: 0.89–1.21, *p* = 0.61), ORR (95% CI: 1.00–1.18, *p* = 0.06), and DCR (95% CI: 0.93–1.05, *p* = 0.68). Erlotinib induced a significantly higher rate of dose reduction (95% CI: 0.13–0.65, *p* = 0.002) and grade 3–5 AEs (95% CI: 0.27–0.71, *p* = 0.0008). In subgroup analysis of AEs, the erlotinib group had a significantly higher rate and severity of skin rash, nausea/vomiting, diarrhea, fatigue and stomatitis.

**Conclusions:**

With equal anti-tumor efficacy and fewer AEs compared with erlotinib, gefitinib is more suitable for treating advanced NSCLC in East Asian patients. Further large-scale, well-designed randomized controlled trials are warranted to confirm our findings.

**Electronic supplementary material:**

The online version of this article (10.1186/s12885-018-4685-y) contains supplementary material, which is available to authorized users.

## Background

In Asia, lung cancer is the most common cancer in men (age-standardized rate [ASR; per 100,000] = 35.2) and the third most common cancer in women (ASR = 12.7). The number of patients with lung cancer has increased rapidly by the year [1, 2]. The discovery and development of therapeutics targeting epidermal growth factor receptor (EGFR), namely tyrosine kinase inhibitors (TKIs), in the past decade was an important clinical advance in non–small cell lung cancer (NSCLC) treatment [3, 4]. Recommended by clinical guidelines, both gefitinib (Iressa) and erlotinib (Tarceva) are now widely accepted as standard-of-care therapy for patients with NSCLC whose tumors harbor activating *EGFR* mutations, especially patients with certain clinical characteristics (Asian descent, female gender, never-smoker, adenocarcinoma) [5–8]. The EGFR TKIs gefitinib and erlotinib both achieve a higher response rate for treating NSCLC in East Asian countries than in the Western countries [9]. However, which EGFR TKI can achieve better efficacy is controversial. In a phase III randomized controlled trial (RCT), Urata reported a higher incidence of grade 3–4 skin rash but less alanine aminotransferase/aspartate aminotransferase elevation in the erlotinib arm. Progression-free survival (PFS), overall survival (OS), and objective response rate (ORR) were similar between the two groups [10]. In another phase III RCT, Yang reported that gefitinib and erlotinib had similar efficacy (PFS, OS, ORR) in NSCLC, with similar toxicities [11]. Some studies have shown that gefitinib has better anti-tumor efficacy or less toxicity for NSCLC [12, 13]. However, other studies have reported opposite results and have suggested that erlotinib is more effective [14, 15].

To resolve this controversy, we conducted a meta-analysis of related studies to compare the anti-tumor efficacy and adverse effects (AEs) of gefitinib and erlotinib for treating East Asian populations with NSCLC.

## Methods

We conducted this meta-analysis according to PRISMA (Preferred Reporting Items for Systematic Review and Meta-Analysis) guidelines.

### Search strategy

The relevant literature was retrieved using the following electronic databases: (1) PubMed; (2) ScienceDirect; (3) The Cochrane Library; (4) Scopus; (5) Web of Science; (6) Embase; (7) Ovid MEDLINE; and (8) Google Scholar. The last search was on February 14, 2018. The following terms were used: “gefitinib”, “erlotinib”, and “Lung cancer”. The complete search we used for PubMed was: (gefitinib [MeSH Terms] OR gefitinib [Text Word] OR IRESSA [Text Word] OR ZDl839 [Text Word]) AND (erlotinib [MeSH Terms] OR erlotinib [Text Word] OR Tarceva [Text Word] OR OSI-774 [Text Word]) AND (lung cancer [MeSH Terms] OR lung cancer [Text Word] OR lung carcinoma [Text Word] OR lung neoplasm [Text Word] OR NSCLC [Text Word]). The references of retrieved articles were also searched for further eligible articles. No language restriction was imposed.

### Selection criteria

Articles that met the following criteria were included: (1) East Asian population with histologically or cytologically confirmed NSCLC based on the Eastern Cooperative Oncology Group; (2) compared gefitinib versus erlotinib; (3) outcomes were PFS, OS, ORR, disease control rate (DCR), and AEs. We excluded reviews without original data, meta-analyses, animal experiments, abstracts only, and studies with duplicated data.

### Data extraction

Two investigators extracted the following data independently: first author, publication year, country, number of participants, participant characteristics (age, sex, stage of cancer, pathological type, line of treatment), anti-tumor efficacy indices (PFS, OS, ORR, DCR), and number of AEs (total AEs, grade 3–5 AEs). A third investigator resolved disagreements on all terms.

### Quality assessment

The quality of RCTs was assessed using the 5-point Jadad scale, which contains questions on three main items: randomization, masking, and accountability of all patients. High-quality studies score ≥ 3 points [16].

The quality of cohort studies was assessed using the Newcastle-Ottawa Scale (NOS, 9 points), which also contains questions on three main items: selection, comparability, and exposure. High-quality studies score 8–9 points; medium-quality studies score 6–7 points [17].

### Statistical analysis

The meta-analysis was conducted using Review Manager (version 5.3, The Nordic Cochrane Centre) and STATA (version 12.0, Stata Corp). Hazard ratios (HR) with 95% confidence intervals (CI) were used to analyze the PFS and OS (HR > 1 favors the erlotinib group; HR < 1 favors the gefitinib group). The HR data were extracted directly from some studies or from Kaplan–Meier curves according to Tierney et al. [18] from other studies. Pooled risk ratios (RR) with 95% CIs were used to analyze the ORR, DCR, and AEs (RR > 1 favors the gefitinib group; RR < 1 favors the erlotinib group). Subgroup analysis of PFS, OS, and ORR was conducted to determine whether the results would change according to *EGFR* mutation status, ethnicity, line of treatment, histology, tumor stage, and study design. Heterogeneity was evaluated using the χ^2^ test and *I*^*2*^ statistic. If *I*^*2*^ > 50% or *p* <  0.1 for the χ^2^ test, reflecting significant heterogeneity, the random-effects model was used; otherwise, the fixed-effects model was used. Publication bias was explored using Begg’s rank correlation and Egger’s linear regression tests. *P* <  0.05 indicated statistical significance.

## Results

### Search results and study quality assessment

**Fig. 1:**
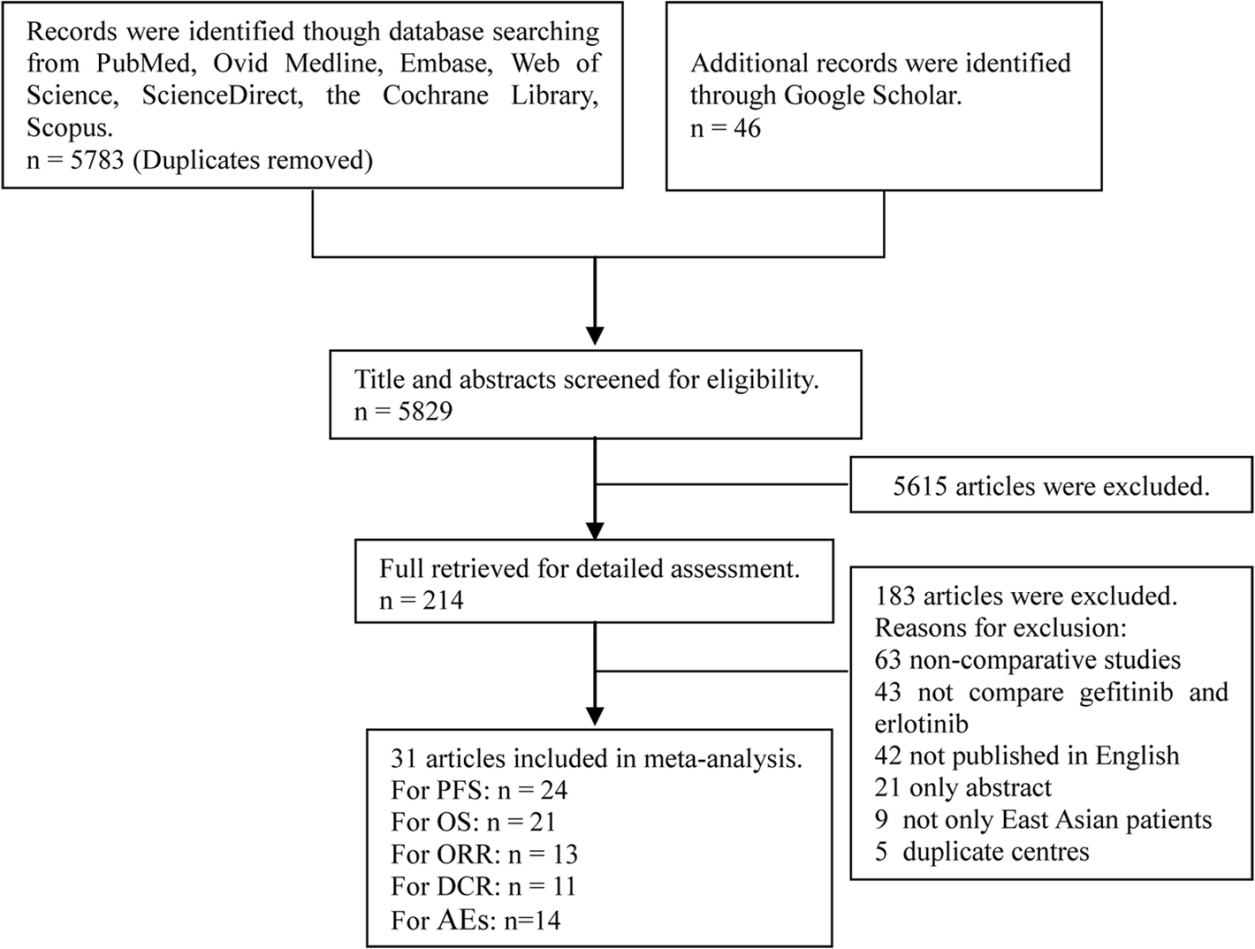
Flow chart of study selection

We initially identified 5829 potentially eligible studies. After screening, 31 studies involving 8054 patients (gefitinib group, 4907 patients; erlotinib group, 3147 patients) were included for the final analysis (Fig. 1) [10–15, 19–43]. Of the 31 studies, three were RCTs and 28 were retrospective studies. Twenty-two studies were of high quality (three RCTs scored 4–5 points, five retrospective studies scored 9 points, 14 retrospective studies scored 8 points) and nine studies were of medium quality (seven retrospective studies scored 7 points, two retrospective studies scored 6 points) (Table 1). Table 2 summarizes the baseline characteristics and main evaluation indices of the included studies.

**Table 1:** Quality assessment of all included studies

Study		Selection	Comparability	Exposure	Randomization	Masking	Accountability of all patients	Quality (score)
Randomized controlled trial								
2012	Kim [26]				★★	★	★	4
2016	Urata [10]				★★	★★	★	5
2017	Yang [11]				★★	★★	★	5
Retrospective study								
2010	Kim [19]	★★★	★★	★★				7
2010	Hotta [20]	★★★★	★★	★★★				9
2010	Hong [21]	★★★	★★	★★				7
2011	Wu [22]	★★★★	★★	★★★				9
2011	Shin [12]	★★★	★★	★★				7
2011	Togashi [23]	★★★★	★★	★★				8
2011	Fan [14]	★★★★	★★	★★				8
2011	Jung [24]	★★★	★★	★				6
2012	Wu [25]	★★★★	★★	★★				8
2012	Suzumura [27]	★★★	★★	★★★				8
2013	Yoshida [28]	★★★★	★★	★★				8
2013	Shao [29]	★★★★	★★	★★★				9
2013	Lee [30]	★★★★	★★	★★				8
2013	Yu [31]	★★★★	★★	★★				8
2014	Lim [32]	★★★★	★★	★★★				9
2014	Sato [13]	★★★★	★★	★★				8
2014	Lin [33]	★★★	★★	★★				7
2014	Ren [34]	★★★★	★★	★★				8
2014	Li [35]	★★★	★★	★★★				8
2014	Takeda [36]	★★★	★★	★				6
2015	Otsuka [37]	★★★★	★★	★★★				9
2015	Song [38]	★★★	★★	★★				7
2015	Koo [39]	★★★★	★★	★				7
2016	Ruan [40]	★★★	★★	★★★				8
2016	Hirano [41]	★★★	★★	★★★				8
2016	Suh [42]	★★★	★★	★★				7
2016	Kashima [43]	★★★	★★	★★★				8
2017	Kuan [15]	★★★★	★★	★★				8

**Table 2:** Characteristics of included studies

Study		Country	Groups	Patients (n)	Median age (year)	Stage	Treatment line	EGFRmutations	Adenocarcinoma (%)	Design	Quality (score)
2010	Kim [19]	Korea	G vs. E	171/171	58/59	IIIb, IV	2, 3	–	86	RS	7
2010	Hotta [20]	Japan	G vs. E	330/209	68/68	II-IV or recurrent	2, 3	–	76	RS	9
2010	Hong [21]	Keroa	G vs. E	20/17	61/67	IIIb, IV	2, 3	–	75	RS	7
2011	Wu [22]	Taiwan	G vs. E	440/276	67/67	IIIb, IV	1 or later	Partial	85	RS	9
2011	Shin [12]	Keroa	G vs. E	100/82	65/65	III, IV	2	Partial	0	RS	7
2011	Togashi [23]	Japan	G vs. E	85/69	65/68	IIIb, IV	1 or later	Partial	82	RS	8
2011	Fan [14]	Taiwan	G vs. E	715/407	–	IIIb, IV	1 or later	Partial	77	RS	8
2011	Jung [24]	Korea	G vs. E	72/51	55/55	IIIb, IV	1 or later	Partial	59	RS	6
2012	Wu [25]	Taiwan	G vs. E	124/100	–	IIIb, IV	1 or later	Partial	100	RS	8
2012	Kim [26]	Keroa	G vs. E	48/48	59/60	IIIb, IV	2	Partial	91	RCT	4
2012	Suzumura [27]	Japan	G vs. E	232/86	67/66	IIIb, IV	–	Partial	95	RS	8
2013	Yoshida [28]	Japan	G vs. E	107/35	64/67	III, IV or recurrent	1 or later	Partial	84	RS	8
2013	Shao [29]	Taiwan	G vs. E	655/329	61/63	IIIb, IV or recurrent	3	–	80	RS	9
2013	Lee [30]	Korea	G vs. E	11/14	49/58	IV	1 or later	Partial	92	RS	8
2013	Yu [31]	China	G vs. E	16/22	54/52	–	3	Partial	100	RS	8
2014	Lim [32]	Korea	G vs. E	121/121	58/58	IIIb, IV	1 or later	All	98	RS	9
2014	Sato [13]	Japan	G vs. E	213/69	66/66	IIIb, IV or recurrent	–	Partial	86	RS	8
2014	Lin [33]	China	G vs. E	57/24	–	IIIb, IV	1	All	59	RS	7
2014	Ren [34]	China	G vs. E	60/142	59/59	IV	1 or later	Partial	66	RS	8
2014	Li [35]	China	G vs. E	53/97	59/59	IIIb, IV	2	Partial	67	RS	8
2014	Takeda [36]	Japan	G vs. E	57/11	69/69	III, IV or recurrent	1 or later	All	99	RS	6
2015	Otsuka [37]	Japan	G vs. E	35/9	70/62	IIIb, IV	1 or later	All	91	RS	9
2015	Song [38]	China	G vs. E	37/65	75/75	IIIb, IV	2 or later	Partial	83	RS	7
2015	Koo [39]	Korea	G vs. E	166/56	–	IV	1, 2, 3	All	87	RS	7
2016	Ruan [40]	China	G vs. E	63/134	59/60	III, IV	–	All	–	RS	8
2016	Hirano [41]	Japan	G vs. E	10/16	71/71	IB-IV or recurrent	–	All	81	RS	8
2016	Urata [10]	Japan	G vs. E	279/280	68/67	IIIb, IV or recurrent	2, 3	Partial	100	RCT	5
2016	Suh [42]	Korea	G vs. E	146/5	65/65	IIIb, IV	1	All	97	RS	7
2016	Kashima [43]	Japan	G vs. E	52/11	68/68	IV	–	All	–	RS	8
2017	Yang [11]	China	G vs. E	128/128	–	IIIb, IV	1, 2	All	96	RCT	5
2017	Kuan [15]	Taiwan	G vs. E	304/63	65/67	IIIb, IV	1	All	–	RS	8

*Abbreviations: G* gefitinib, *E* erlotinib, *EGFR* epidermal growth factor receptor, *RS* retrospective study, *RCT* randomized controlled trial, −: not available

### Anti-tumor efficacy

We assessed anti-tumor efficacy between the gefitinib and erlotinib groups based on PFS, OS, ORR, and DCR.

**Fig. 2:**
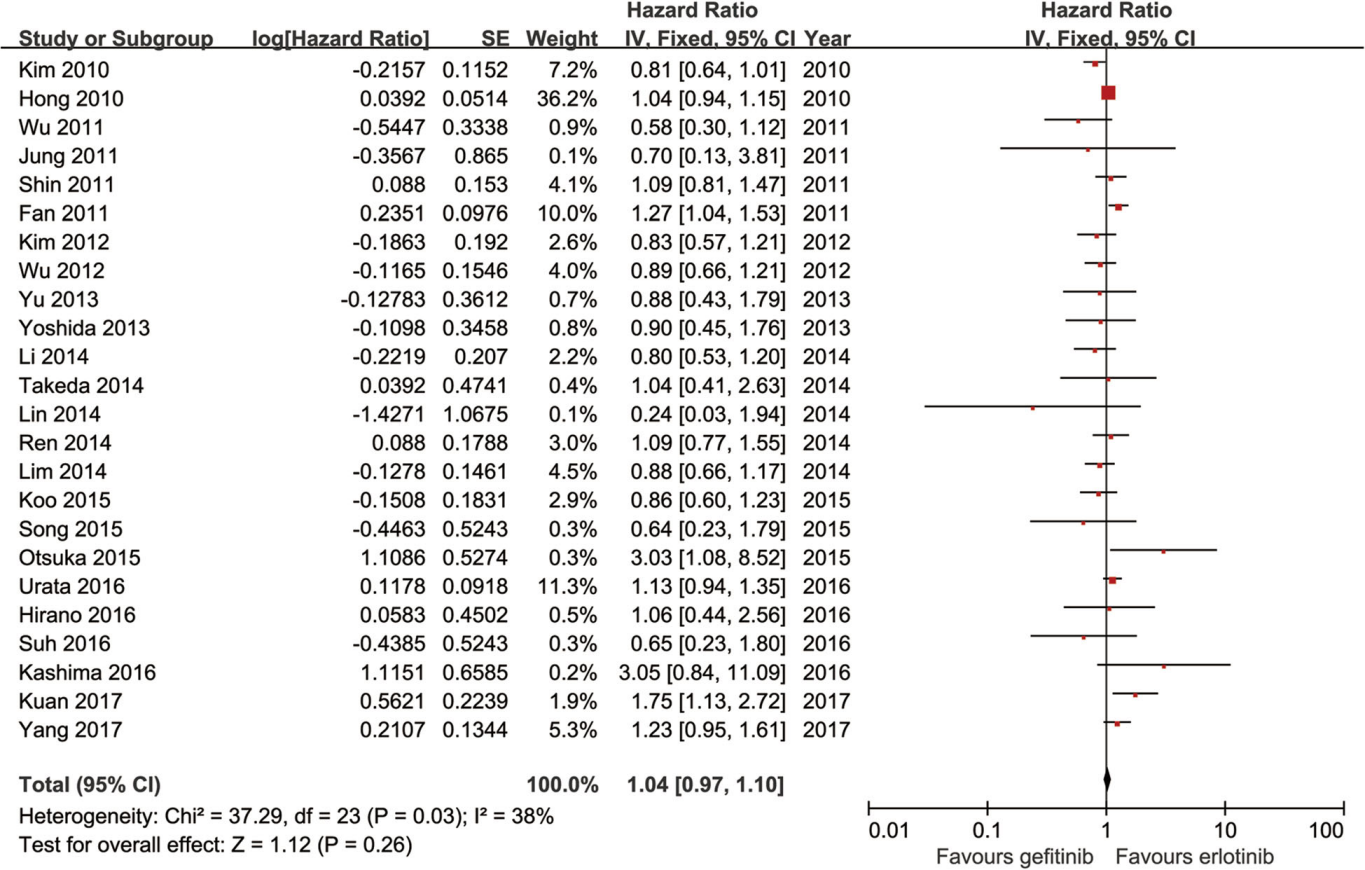
Forest plot of HR of PFS associated with gefitinib versus erlotinib

Twenty-four studies compared PFS (heterogeneity: *p* = 0.03, *I*^2^ = 38%). No significant difference was found between the two groups (95% CI: 0.97–1.10, *p* = 0.26; Fig. 2).

**Fig. 3:**
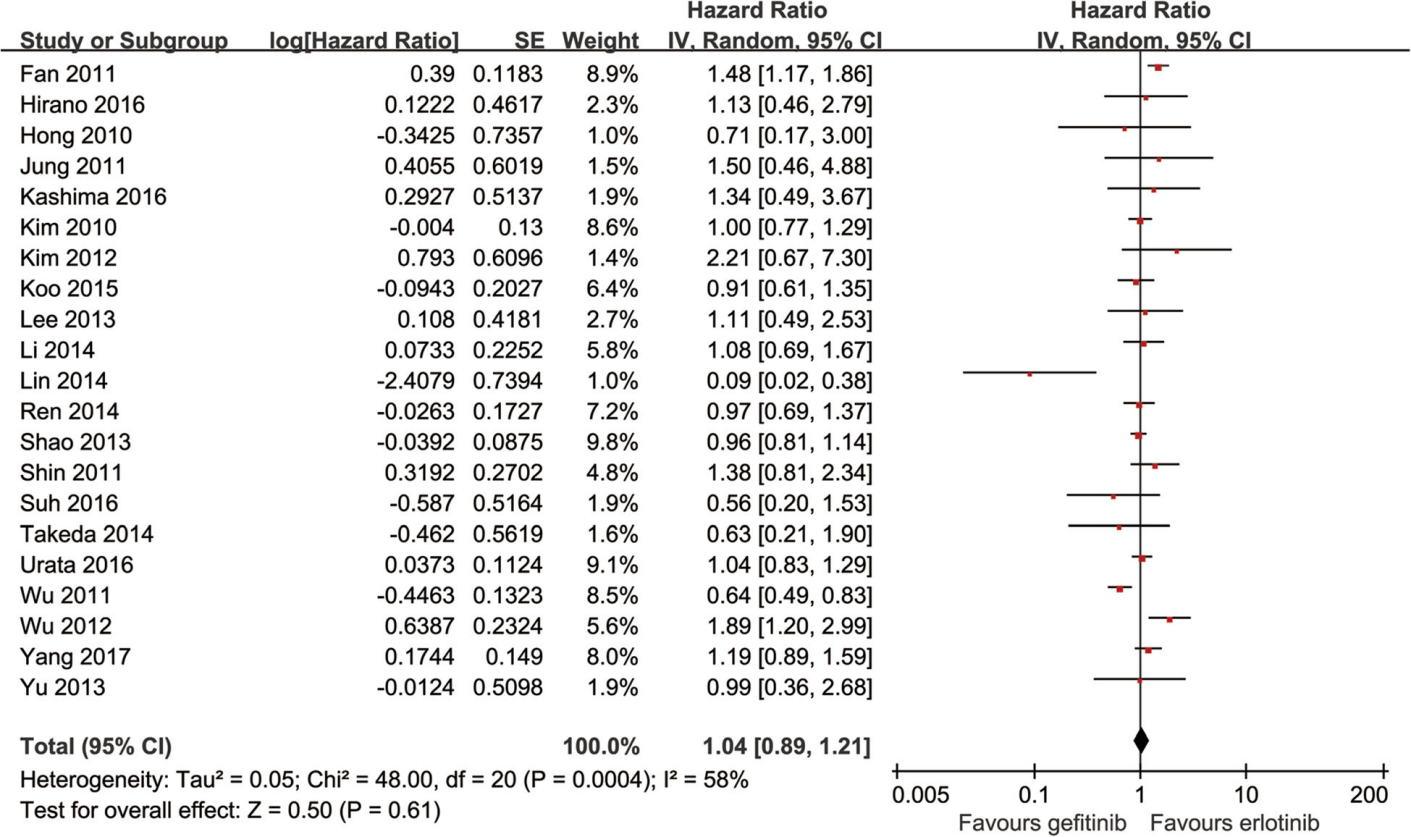
Forest plot of HR of OS associated with gefitinib versus erlotinib

Twenty-one studies compared OS (heterogeneity: *p* = 0.0004, *I*^2^ = 58%). No significant difference was found between the two groups (95% CI: 0.89–1.21, *p* = 0.61; Fig. 3).

**Fig. 4:**
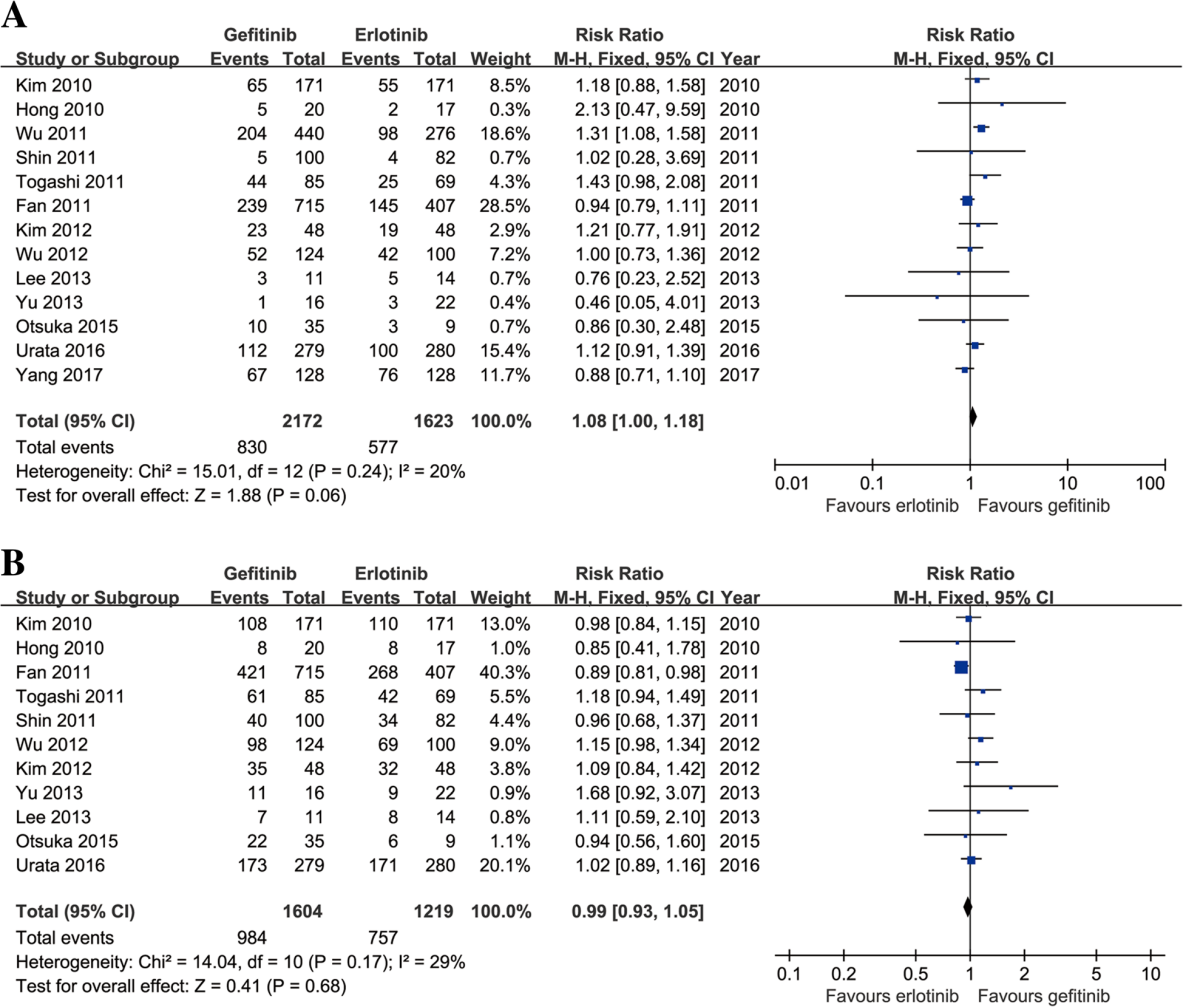
Forest plots of RR of ORR (**a**) and DCR (**b**) associated with gefitinib versus erlotinib

Thirteen studies compared ORR (heterogeneity: *p* = 0.24, *I*^2^ = 20%). No significant difference was found between the two groups (95% CI: 1.00–1.18, *p* = 0.06; Fig. 4a).

Eleven studies compared DCR (heterogeneity: *p* = 0.17, *I*^2^ = 29%). No significant difference was found between the two groups (95% CI: 0.93–1.05, *p* = 0.68; Fig. 4b).

### Toxicity

We compared toxicity between the gefitinib and erlotinib groups based on total AEs, grade 3–5 AEs, and subgroup analysis of the 10 most reported AEs.

**Fig. 5:**
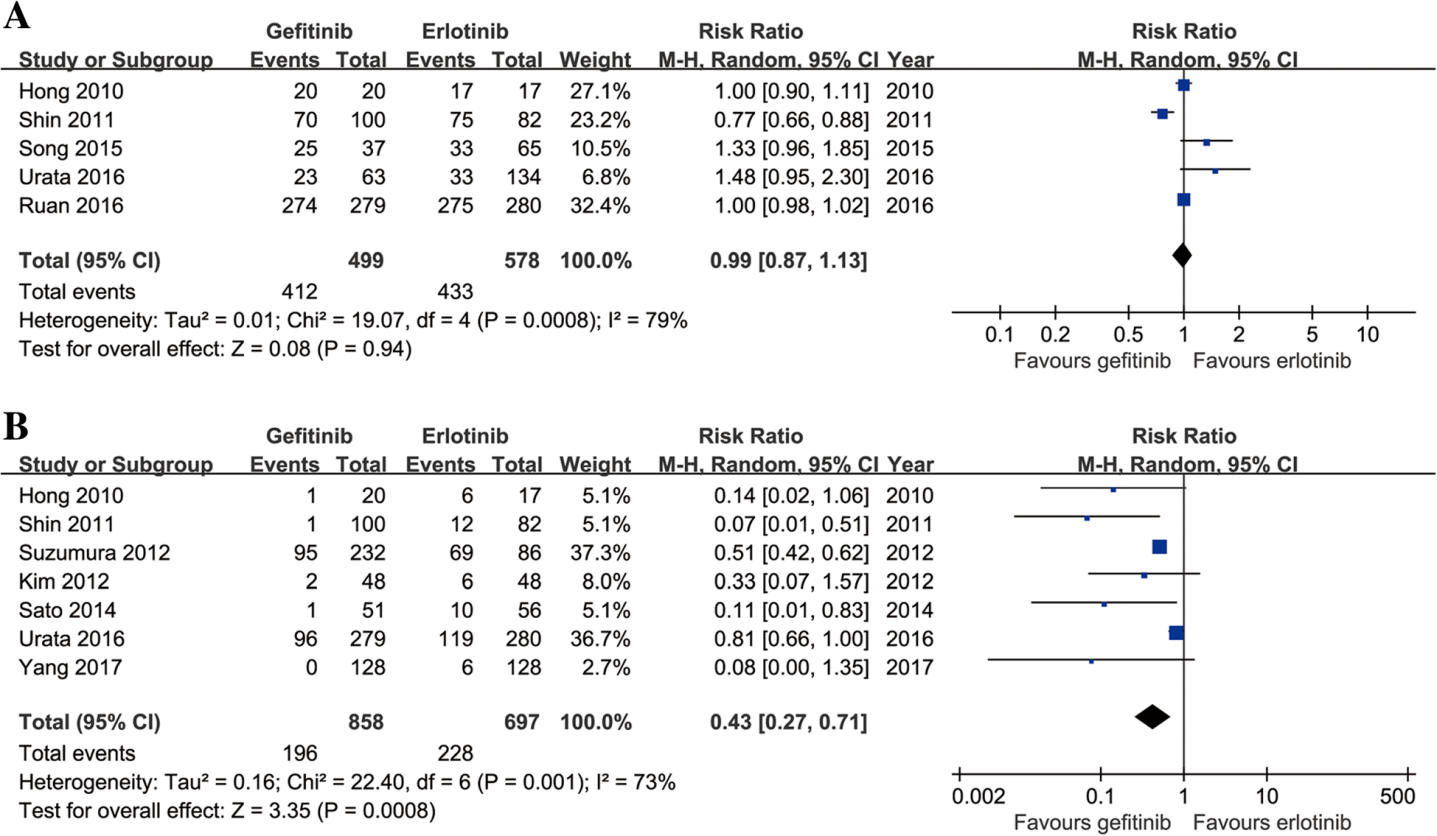
Forest plots of RR of all-grade AEs (**a**) and grade 3–5 AEs (**b**) associated with gefitinib versus erlotinib

Five studies compared total AEs (heterogeneity: *p* = 0.0007, *I*^2^ = 79%). No significant difference was found between the two groups (95% CI: 0.87–1.13, *p* = 0.94; Fig. 5a).

**Fig. 6:**
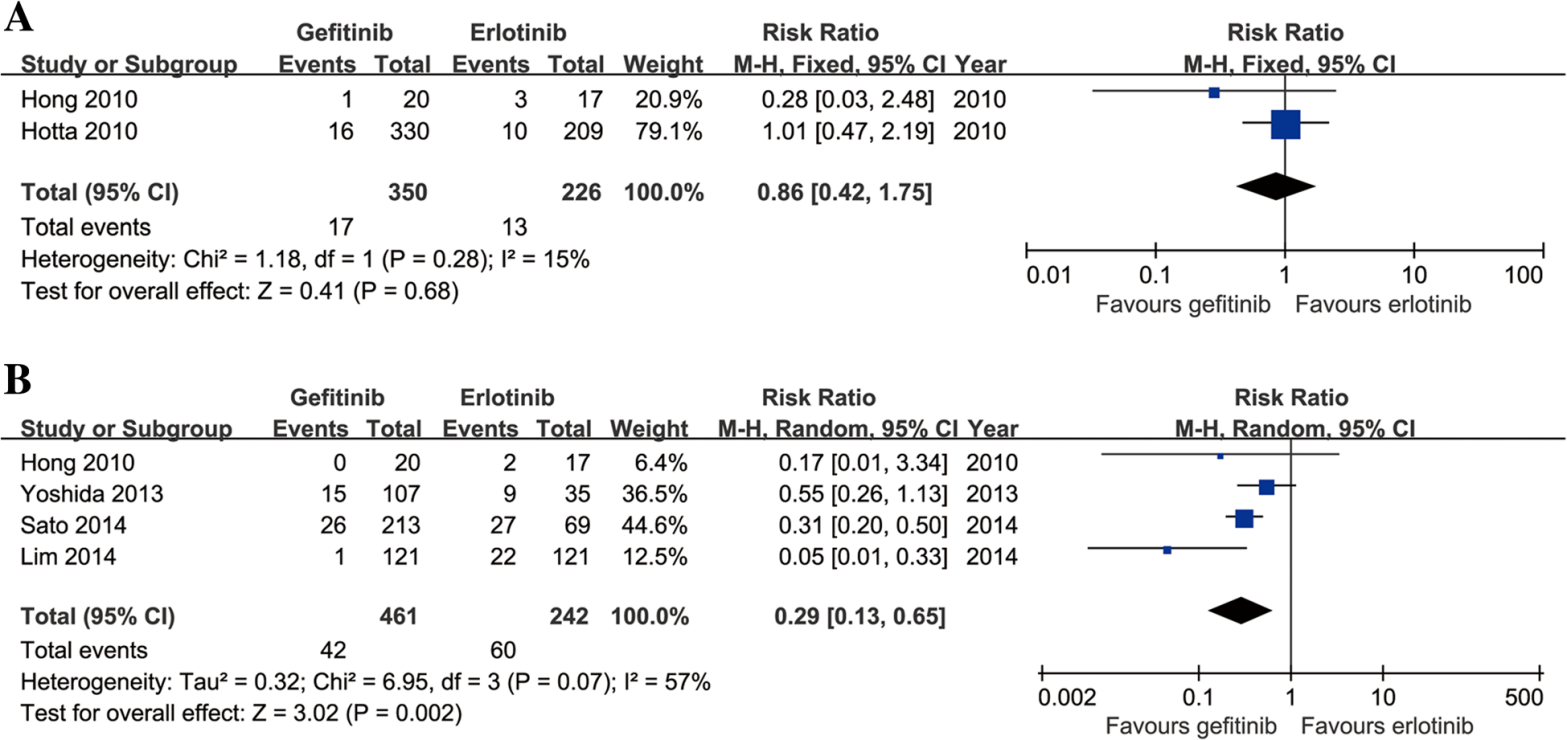
Forest plots of RR of drug discontinuations (**a**) and drug reductions (**b**) associated with gefitinib versus erlotinib

Seven studies compared grade 3–5 AEs (heterogeneity: *p* = 0.001, *I*^2^ = 73%). The gefitinib group had a significantly lower incidence rate of grade 3–5 AEs than the erlotinib group (95% CI: 0.27–0.71, *p* = 0.0008; Fig. 5b). Some patients had drug discontinuations/reductions due to the occurrence of serious AEs. Two studies compared drug discontinuations; there was no significant difference between the two groups (95% CI: 0.40–1.80, *p* = 0.68; Fig. 6a). Four studies compared drug reductions; the erlotinib group had more drug reductions (95% CI: 0.13–0.65, *p* = 0.002; Fig. 6b).

In subgroup analysis of the 10 most reported AEs (skin rash, diarrhea, nausea/vomiting, fatigue, anorexia, interstitial lung disease, stomatitis, elevated liver enzymes, infection, neutropenia), the results for all-grade AEs showed no significant differences in anorexia, interstitial lung disease, elevated liver enzymes, infection, neutropenia and nausea/vomiting between the two groups. For all-grade AEs, erlotinib induced significantly higher rates of skin rash (95% CI: 0.74–0.94, *p* = 0.003), diarrhea (95% CI: 0.73–0.95, *p* = 0.005), fatigue (95% CI: 0.23–0.95, *p* = 0.04), and stomatitis (95% CI: 0.15–0.54, *p* = 0.0001) (Table 3). The results for grade 3–5 AEs showed no significant differences in anorexia, interstitial lung disease, elevated liver enzymes, infection, and neutropenia between the two groups. For grade 3–5 AEs, erlotinib induced significantly higher rates of skin rash (95% CI: 0.12–0.41, *p* < 0.00001), diarrhea (95% CI: 0.29–0.74, *p* = 0.001), nausea/vomiting (95% CI: 0.11–0.49, *p* = 0.0001), fatigue (95% CI: 0.09–0.87, *p* = 0.03), and stomatitis (95% CI: 0.08–0.99, *p* = 0.05) (Table 4).

**Table 3:** Top 10 adverse effects (all grade) associated with gefitinib versus erlotinib

Adverse effects	Gefitinib group (event/total)	Erlotinib group (event/total)	RR (95% CI)	*P* value	Heterogeneity	
					*I*^*2*^ (%)	*P* value
Skin rash	673/1099	650/944	0.83 (0.74–0.94)	0.003	68	0.0009
Diarrhea	298/999	273/745	0.83 (0.73–0.95)	0.005	47	0.06
Nausea/Vomiting	107/639	139/531	0.71 (0.32–1.57)	0.4	74	0.002
Fatigue	124/639	149/531	0.47 (0.23–0.95)	0.04	81	< 0.0001
Anorexia	53/403	40/310	0.98 (0.40–2.42)	0.97	78	0.001
Interstitial lung disease	35/949	19/723	1.38 (0.78–2.44)	0.26	0	0.65
Stomatitis	12/260	29/169	0.29 (0.15–0.54)	0.0001	24	0.27
Elevated liver enzymes	366/931	264/680	1.16 (0.85–0.1.56)	0.35	61	0.04
Infection	45/686	23/466	1.53 (0.93–2.51)	0.1	23	0.27
Neutropenia	61/399	51/379	1.19 (0.85–1.66)	0.32	0	0.55

**Table 4:** Top 10 adverse effects (grade 3–5) associated with gefitinib versus erlotinib

Grade 3–5 Adverse effects	Gefitinib group (event/total)	Erlotinib group (event/total)	RR (95% CI)	*P* value	Heterogeneity	
					*I*^*2*^ (%)	*P* value
Skin rash	72/999	163/745	0.22 (0.12–0.41)	< 0.00001	73	0.0006
Diarrhea	31/892	38/710	0.46 (0.29–0.74)	0.001	0	0.46
Nausea/Vomiting	8/639	27/531	0.23 (0.11–0.49)	0.0001	20	0.29
Fatigue	18/639	40/531	0.28 (0.09–0.87)	0.03	74	0.02
Anorexia	3/403	4/310	0.25 (0.06–1.04)	0.06	NA	NA
Interstitial lung disease	7/619	3/514	1.05 (0.27–4.06)	0.95	17	0.3
Stomatitis	3/260	8/169	0.28 (0.08–0.99)	0.05	24	0.27
Elevated liver enzymes	80/652	23/400	1.50 (0.97–2.31)	0.07	0	0.64
Infection	9/454	7/380	1.12 (0.46–2.69)	0.8	20	0.28
Neutropenia	2/399	3/379	0.67 (0.11–3.97)	0.66	NA	NA

### Subgroup analysis

To determine whether the anti-tumor efficacy of gefitinib versus erlotinib was consistent across subgroups, the pooled efficacy for PFS, OS, and ORR was estimated within each category of the following classification variables: country, tumor stage, histology, line of treatment, *EGFR* mutation status, and study design. All subgroup differences were not statistically significant in terms of PFS, OS, and ORR between the gefitinib and erlotinib groups (Table 5).

**Table 5:** Subgroup analysis for progression-free survival, overall survival and objective response rate

Group	PFS				OS				ORR			
	No.of studies	HR (95% CI)	*P*	*I*^*2*^ (%)	No.of studies	RR (95% CI)	*P*	*I*^*2*^ (%)	No.of studies	RR (95% CI)	*P*	*I*^*2*^ (%)
Total	24	1.04 (0.97–1.10)	0.26	38	21	1.04 (0.89–1.21)	0.61	58	13	1.08 (1.00–1.18)	0.06	20
Nation												
Keroa	8	0.89 (0.78–1.02)	0.09	18	8	1.03 (0.85–1.23)	0.79	0	5	1.18 (0.94–1.49)	0.16	0
China	6	1.05 (0.88–1.25)	0.63	20	5	0.92 (0.62–1.36)	0.67	67	2	0.87 (0.70–1.08)	0.21	0
Japan	6	1.15 (0.98–1.36)	0.09	20	4	1.04 (0.84–1.27)	0.74	0	3	1.18 (0.98–1.41)	0.08	0
Taiwan	4	1.09 (0.77–1.54)	0.62	74	4	1.12 (0.75–1.67)	0.59	90	3	1.07 (0.86–1.35)	0.54	71
Tumor stage												
IIIb-IV	22	1.04 (0.98–1.10)	0.23	40	18	1.08 (0.92–1.26)	0.34	53	12	1.09 (1.00–1.18)	0.05	24
I-IV	2	0.77 (0.39–1.51)	0.45	25	3	0.54 (0.18–1.63)	0.27	80	1	0.46 (0.05–4.01)	0.48	NA
History												
Non-squamous	13	1.04 (0.96–1.14)	0.88	51	11	1.06 (0.86–1.31)	0.58	68	9	1.08 (0.99–1.17)	0.09	42
Squamous included	10	1.02 (0.94–1.12)	0.6	11	9	0.98(0.86–1.13)	0.81	48	4	1.19 (0.81–1.77)	0.38	0
Unclear	1	3.05 (0.84–11.09)	0.09	NA	1	1.34 (0.49–3.67)	0.57	NA				
Treatment line												
First line included	14	1.09 (0.98–1.20)	0.11	46	11	0.97 (0.72–1.30)	0.82	77	7	1.06 (0.90–1.25)	0.52	52
Second line or later	8	1.01 (0.93–1.08)	0.89	22	8	1.02 (0.91–1.14)	0.78	0	6	1.15 (0.98–1.35)	0.08	0
First line only	3	0.89 (0.32–2.49)	0.82	66	2	0.24 (0.04–1.43)	0.12	75				
Second line only	3	0.93 (0.76–1.14)	0.5	0	2	1.25 (0.90–1.73)	0.19	0	2	1.18 (0.76–1.82)	0.47	0
Third line only	1	0.88 (0.43–1.79)	0.72	NA	2	0.96 (0.81–1.14)	0.47	0	1	0.46 (0.05–4.01)	0.48	NA
Unclear	2	1.48 (0.72–3.08)	0.29	43	2	1.22 (0.62–2.39)	0.56	0				
EGFR mutation												
Partial mutation	11	1.02 (0.91–1.15)	0.68	21	11	1.15 (0.91–1.45)	0.24	68	9	1.10 (1.00–1.21)	0.05	21
All mutation	9	1.11 (0.90–1.36)	0.33	50	7	0.82 (0.54–1.25)	0.36	59	2	0.88 (0.71–1.09)	0.24	0
Unclear	4	0.98 (0.76–1.26)	0.88	57	3	0.97 (0.84–1.13)	0.67	0	2	1.22 (0.92–1.62)	0.18	2
Study design												
Retrospective study	21	1.02 (0.95–1.09)	0.37	40	18	1.01 (0.84–1.21)	0.92	63	10	1.10 (1.00–1.22)	0.06	19
RCT	3	1.11 (0.96–1.27)	0.15	32	3	1.11 (0.93–1.32)	0.25	0	3	1.04 (0.90–1.20)	0.62	36

*Abbreviations: PFS* progression-free survival, *OS* overall survival, *ORR* objective response rate, *ORR* objective response rate, *HR* hazard ratio, *RR* relative risk, *RCT* randomized controlled trial, *NA* not available

### Sensitivity analysis

**Fig. 7:**
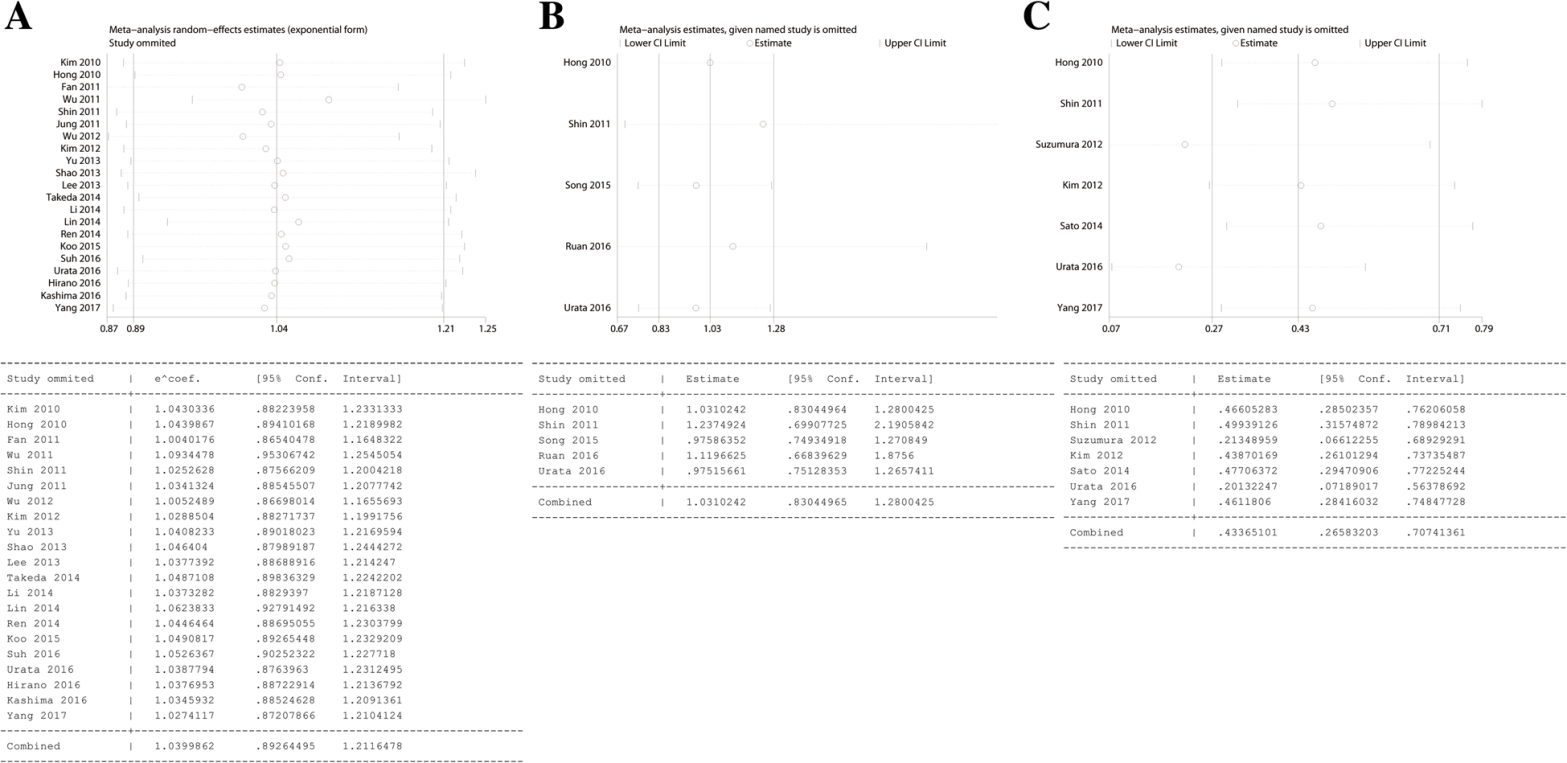
Meta-based influence analysis for comparisons of OS (**a**), total AEs (**b**) and grade 3–5 AEs (**c**)

Significant heterogeneity was found in the analysis of OS, total AEs and grade 3–5 AEs. The influence of each study on the pooled results was evaluated to evaluate stability and sensitivity. The results suggested that the outcomes of OS, total AEs and grade 3–5 AEs were reliable and stable (Fig. 7).

### Cumulative meta-analysis

Analyses of PFS (Additional file 1: Figure S1), OS (Additional file 2: Figure S2), ORR (Additional file 3: Figure S3), DCR (Additional file 4: Figure S4) and total AEs (Additional file 5: Figure S5) demonstrated that the RRs of the final results became robust within a narrow range and remained not significant as publication years increased and as recent high-quality studies were included. After inclusion of Shin et al.’s study [12], the RR and 95% CI for grade 3–5 AEs decreased to < 1 and became stable (Additional file 6: Figure S6). Although there was no significantly reduced risk in ORR, clear evidence showed that the confidence interval was becoming narrow, and trended toward significance (favors gefitinib).

### Publication bias

**Fig. 8:**
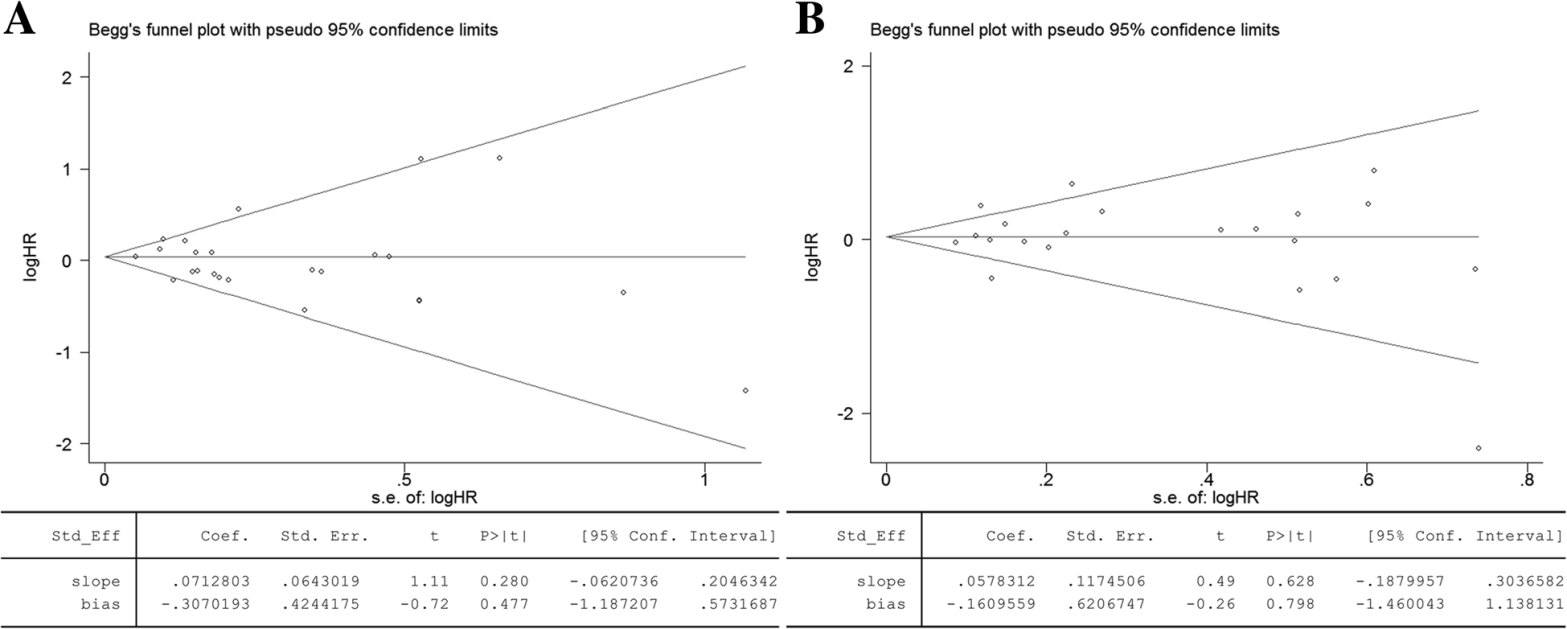
Begg’s and Egger’s tests for comparisons of PFS (**a**) and OS (**b**)

There was no evidence of publication bias for PFS (Begg’s test *p* = 0.585; Egger’s test *p* = 0.477, Fig. 8a) and OS (Begg’s test *p* = 0.880; Egger’s test *p* = 0.798, Fig. 8b).

## Discussion

Gefitinib and erlotinib are two similar small molecules with different binding capabilities and pharmacokinetic and pharmacodynamic properties related to their differing molecular structures [44–46]. Whether the differences between these first-generation EGFR TKIs can cause different anti-tumor efficacy is controversial [10, 11, 47]. By analyzing 31 high-quality studies, we directly compared the anti-tumor efficacy and safety of gefitinib and erlotinib for treating NSCLC [10–15, 19–43]. Our meta-analysis provides the most current medical evidence and shows that anti-tumor efficacy (PFS, OS, ORR, DCR) is comparable between gefitinib and erlotinib for treating East Asian patients with NSCLC. Subgroup analysis according to country, tumor stage, histology, line of treatment, EGFR mutation, and study design did not change the results. However, erlotinib toxicity was significantly greater than that of gefitinib, especially in all-grade/grade 3–4 skin rash, nausea/vomiting, fatigue, and stomatitis.

The greater drug toxicity is an critical problem regarding erlotinib. In our analysis, we found high incidences of drug reduction, skin rash, diarrhea, nausea/vomiting, fatigue, and stomatitis in the erlotinib arm. Although it might not decrease survival time, it greatly reduces patients’ quality of life [48, 49]. We believe there are two reasons for these results: (1) the oral dose of erlotinib (150 mg/day) was closer to the maximum tolerated dose (150 mg/day) as compared with gefitinib (oral dose, 250 mg/day; maximum tolerated dose, 600 mg/day) [50, 51]; (2) The two EGFR TKIs have different pharmacokinetics. After absorption, more gefitinib accumulates in tumor tissue than in plasma; the opposite is true for erlotinib [52]. In the published literature, more severe AEs have been reported in East Asian patients as compared with patients from Europe and America [9, 53]. Interstitial lung disease is one of the most important AEs, and can cause worse prognosis and increased risk of death [54]. However, our analysis and other published studies show that most cases of interstitial lung disease are reported in East Asian populations and that it is rare in Western populations. This might be attributed to the smaller physiques of Asians in general. In a retrospective study, Yeo reduced the erlotinib dose to 25 mg/day and achieved similar or even better prognosis as compared with the standard dose [55]. Other retrospective studies have reported similar results [13, 56–58]. Accordingly, we suggest that individualized drug dose based on weight or body surface area might be more appropriate than a fixed oral dose for treating advanced NSCLC. More large-sample, well-designed RCTs are needed to confirm the best dose of gefitinib and erlotinib for East Asian patients with advanced NSCLC.

Almost all of the included studies did not show any differences in all anti-tumor efficacy indices, which formed the basis of our results. Only one study reported an unfavorable result for erlotinib, with both lower PFS and OS, which might relate to the erlotinib group having more patients with non-adenocarcinoma NSCLC as based on government regulations [14]. Our results also showed a trend for prolonged median PFS (gefitinib group, 7.1 months vs. 4.9 months; erlotinib group, 7.7 months vs. 3.4 months) and OS (gefitinib group, 19.1 months vs. 14.0 months; erlotinib group, 15.5 months vs. 12.7 months) in patients with adenocarcinoma as compared with squamous-included NSCLC. However, no difference was found between gefitinib and erlotinib in this subgroup.

In the *EGFR* mutation status subgroup, we also found no difference between the anti-tumor efficacy of gefitinib and erlotinib. However, our results indirectly prove that both gefitinib and erlotinib are more suitable for treating *EGFR* mutation–positive NSCLC. Both median PFS (gefitinib group, 11.4 months vs. 4.9 months; erlotinib group, 9.6 months vs. 3.1 months) and OS (gefitinib group, 22.6 months vs. 16.0 months; erlotinib group, 20.9 months vs. 12.0 months) were longer in the *EGFR* mutation–positive subgroup than in the partial *EGFR* mutation–positive subgroup. Accordingly, we observed that the proportion of *EGFR* mutations increased by the year in EGFR TKI treatment (Table 1). Multiple *EGFR* mutation isoforms (exon 19, exon 21, others) were found, although the isoform most susceptible to gefitinib or erlotinib remains unclear. A phase III RCT compared gefitinib and erlotinib treatment in *EGFR* mutation–positive NSCLC and found significantly higher RR and longer median OS for patients with *EGFR* exon 19 mutations than for patients with *EGFR* exon 21 mutations following erlotinib or gefitinib treatment. However, no difference was found between gefitinib and erlotinib for both mutations [11]. Another RCT involving more *EGFR* mutation isoforms (exon 19, exon 21, T790 M) reported similar results [10]. However, Kuan suggested that erlotinib is associated with significantly longer PFS and lower risk of progression than gefitinib in patients with *EGFR* exon 19 deletions [15]. Limited by the quantity of published studies and included patients, further large-sample, well-designed RCTs focusing on single *EGFR* mutations are warranted to identify the best EGFR TKIs.

The line of treatment in which EGFR TKIs should be used in NSCLC remains controversial. Mainstream thinking considers EGFR TKIs second-line or later treatment after chemotherapy failure or first-line treatment for patients unable to tolerate chemotherapy. However, Table 1 shows that an increasing number of studies have used gefitinib and erlotinib as first-line treatment for advanced NSCLC [15, 33, 42]. However, no differences were found for PFS, OS, and ORR between gefitinib and erlotinib in each line of treatment subgroup. Wu et al. conducted a phase III RCT and suggested that first-line erlotinib can significantly improve PFS as compared to gemcitabine+cisplatin in patients with *EGFR* mutation–positive NSCLC [59]. Another phase III RCT suggested that PFS is significantly longer with gefitinib treatment in patients with mutation-positive NSCLC as compared with carboplatin+paclitaxel [60]. Several other high-quality RCTs have reported similar results [61–63]. Based on these positive results, the US Food and Drug Administration approved gefitinib as first-line treatment for *EGFR* mutation–positive NSCLC [64]. In the 2017 National Comprehensive Cancer Network (NCCN) guideline on NSCLC, both gefitinib and erlotinib are suggested as first-line treatment for *EGFR* mutation–positive NSCLC [65].

Several limitations should considered when interpreting our results. First, only high-quality studies published in English were included, which might result in language bias. Second, only three RCTs were included, which would weaken the quality of the results. Third, there was significant heterogeneity for some comparisons (OS and total/grade 3–5 AEs), which would weaken the reliability of these results. Fourth, the type and rate of *EGFR* mutations differed between the included studies, which might increase heterogeneity and weaken the quality of the results. Fifth, we obtained data from only three East Asian countries (China [Mainland and Taiwan], Japan, Korea), which might reduce the representativeness of the study. Sixth, quality of life and survival time are two equally important evaluating indicators for a treatment. Quality of life cannot simply be replaced by the number of AEs. However, the included studies did not compare quality of life between treatment with the two EGFR TKIs. Accordingly, we suggest that quality of life be considered an essential indicator in future drug evaluation studies.

## Conclusion

Our results show that both gefitinib and erlotinib are effective for treating advanced NSCLC in East Asian patients, with comparable PFS, OS, ORR, and DCR. Erlotinib induces a significantly higher rate and severity of skin rash, nausea/vomiting, fatigue, and stomatitis, which might cause a higher rate of dose reduction. Therefore, we suggest that individualized drug dose based on weight or body surface area might be more appropriate than a fixed oral dose for both agents in treating East Asian patients with advanced NSCLC. However, due to the inherent limitations of our meta-analysis, more large-scale, high-quality RCTs are warranted to confirm this conclusion.

## Additional files

Additional file 1: **Figure S1.** Cumulative meta-analysis related to PFS associated with gefitinib versus erlotinib. (TIFF 1895 kb)

Additional file 2: **Figure S2.** Cumulative meta-analysis related to OS associated with gefitinib versus erlotinib. (TIFF 1885 kb)

Additional file 3: **Figure S3.** Cumulative meta-analysis related to ORR associated with gefitinib versus erlotinib. (TIFF 1498 kb)

Additional file 4: **Figure S4.** Cumulative meta-analysis related to DCR associated with gefitinib versus erlotinib. (TIF 1379 kb)

Additional file 5 **Figure S5.** Cumulative meta-analysis related to total AEs associated with gefitinib versus erlotinib. (TIFF 999 kb)

Additional file 6: **Figure S6.** Cumulative meta-analysis related to grade 3–5 AEs associated with gefitinib versus erlotinib. (TIFF 1104 kb)

## Abbreviations

AEs: Adverse effects
ASR: Age-standardized rate
CI: Confidence interval
DCR: Disease control rate
EGFR TKIs: Epidermal growth factor receptor tyrosine kinase inhibitors
HR: Hazard ratios
NOS: Newcastle-Ottawa Scale
NSCLC: Non-small cell lung cancer
ORR: Objective response rate
OS: Overall survival
PFS: Progression-free survival
PRISMA: Preferred Reporting Items for Systematic Review and Meta-Analysis
RR: Risk ratios
