# Supplementary figures and images for: Gefitinib provides similar effectiveness and improved safety than erlotinib for east Asian populations with advanced non–small cell lung cancer: a meta-analysis
Source: BMC Cancer. 2018 Aug 2;18:780. doi: 10.1186/s12885-018-4685-y (PMC6090934; doi:10.1186/s12885-018-4685-y)

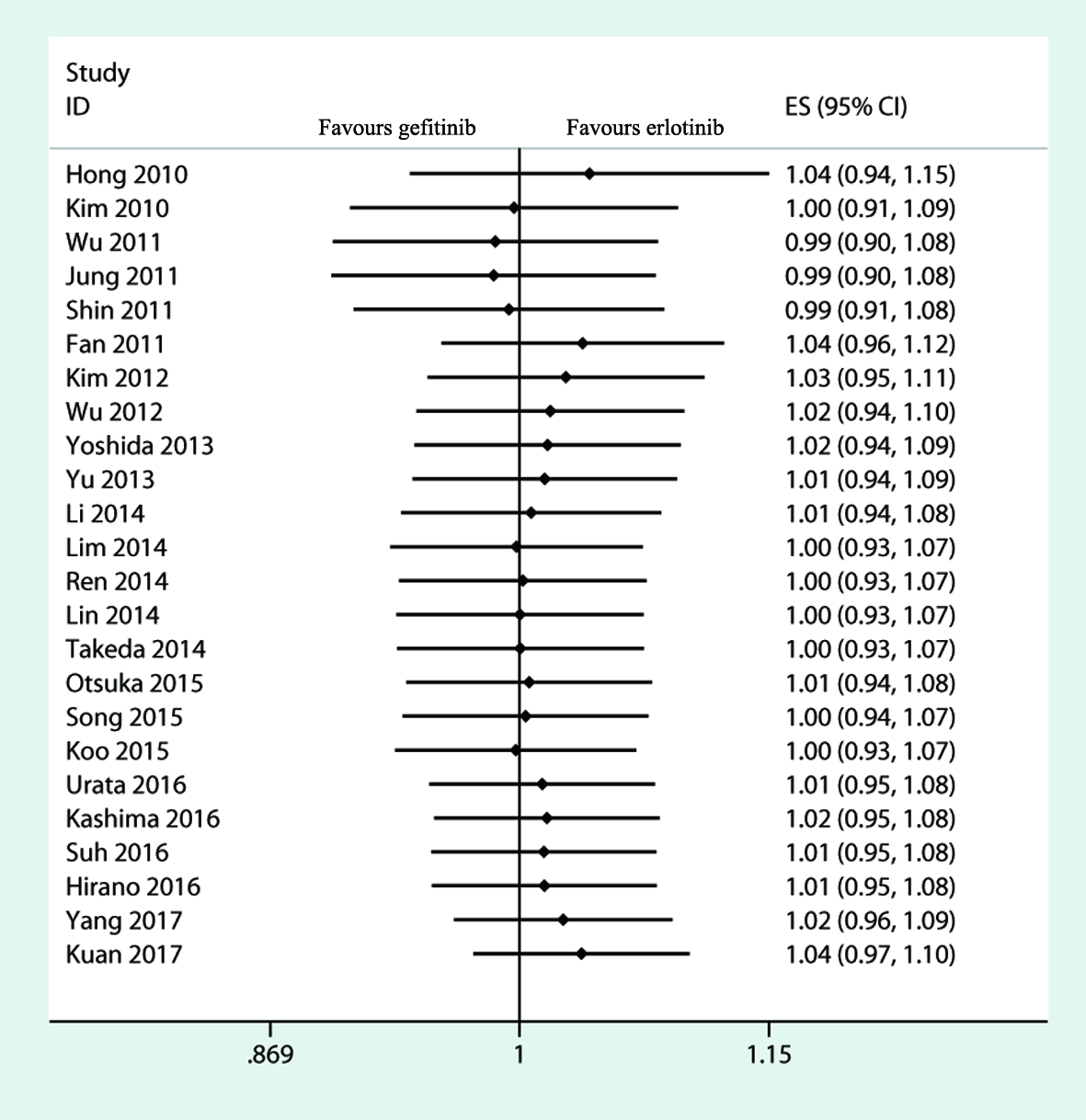

Supplement: Supplementary file 1 — Figure S1. Cumulative meta-analysis related to PFS associated with gefitinib versus erlotinib. (TIFF 1895 kb) [file 12885_2018_4685_MOESM1_ESM.tiff]

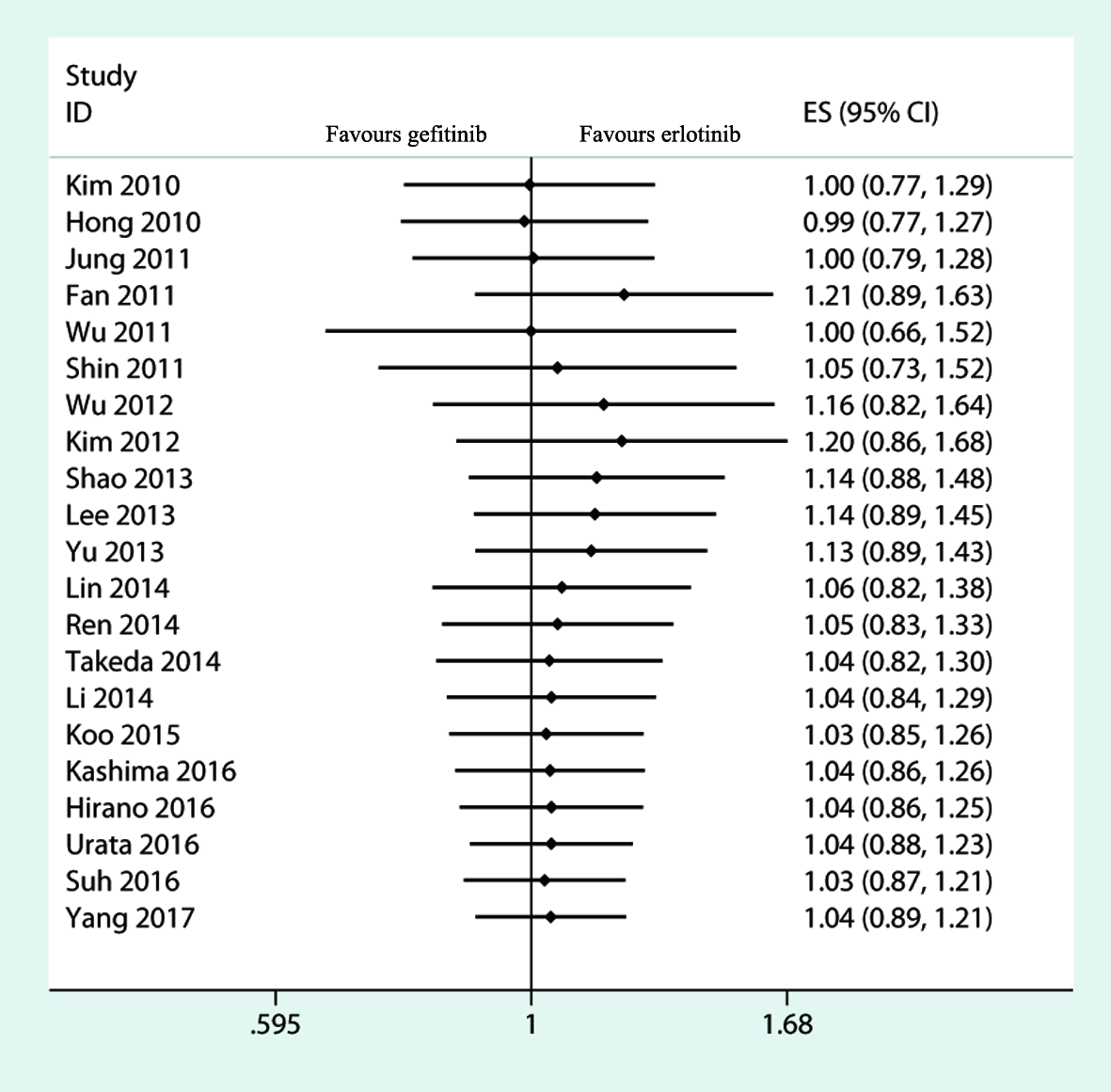

Supplement: Supplementary file 2 — Figure S2. Cumulative meta-analysis related to OS associated with gefitinib versus erlotinib. (TIFF 1885 kb) [file 12885_2018_4685_MOESM2_ESM.tiff]

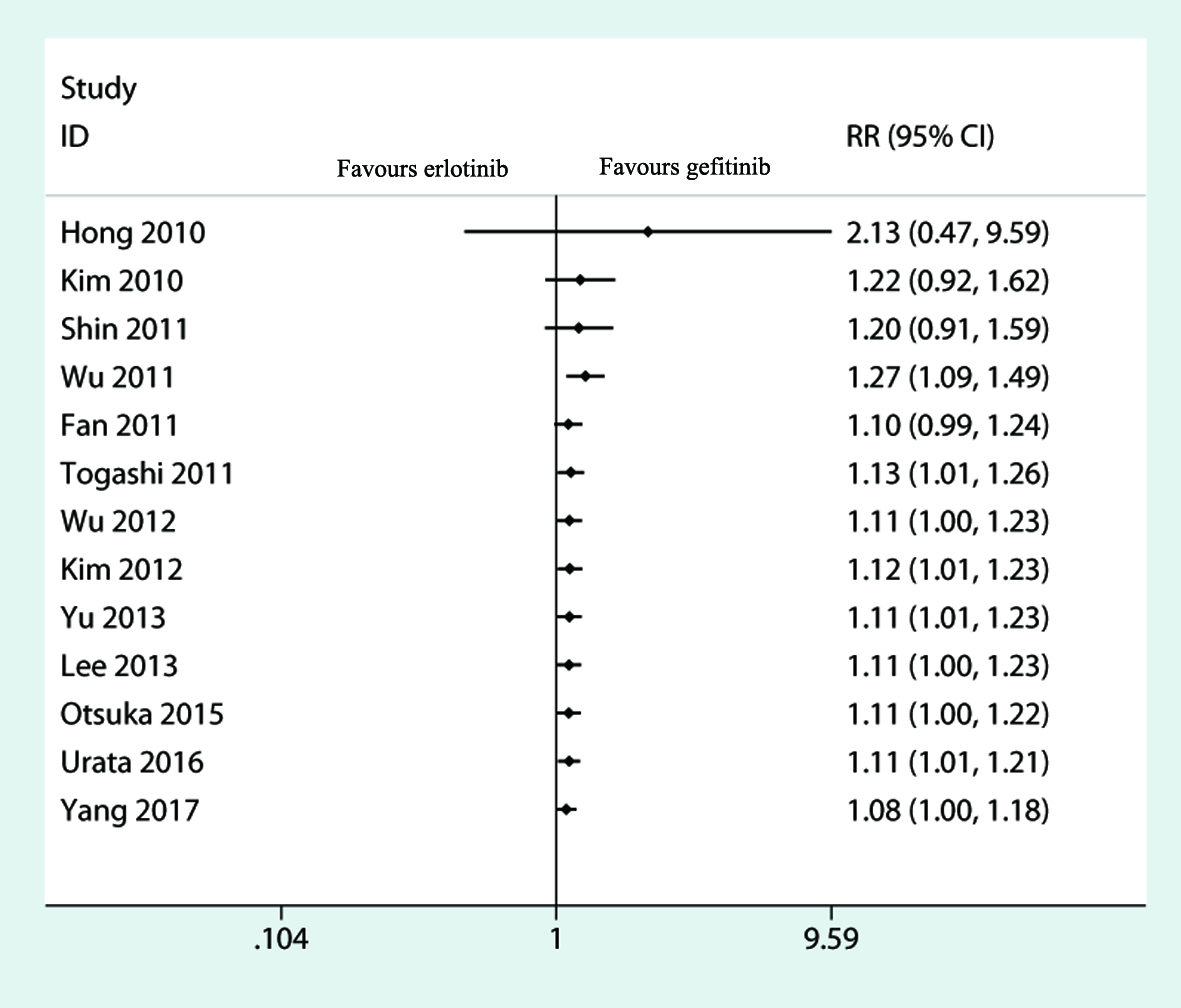

Supplement: Supplementary file 3 — Figure S3. Cumulative meta-analysis related to ORR associated with gefitinib versus erlotinib. (TIFF 1498 kb) [file 12885_2018_4685_MOESM3_ESM.tiff]

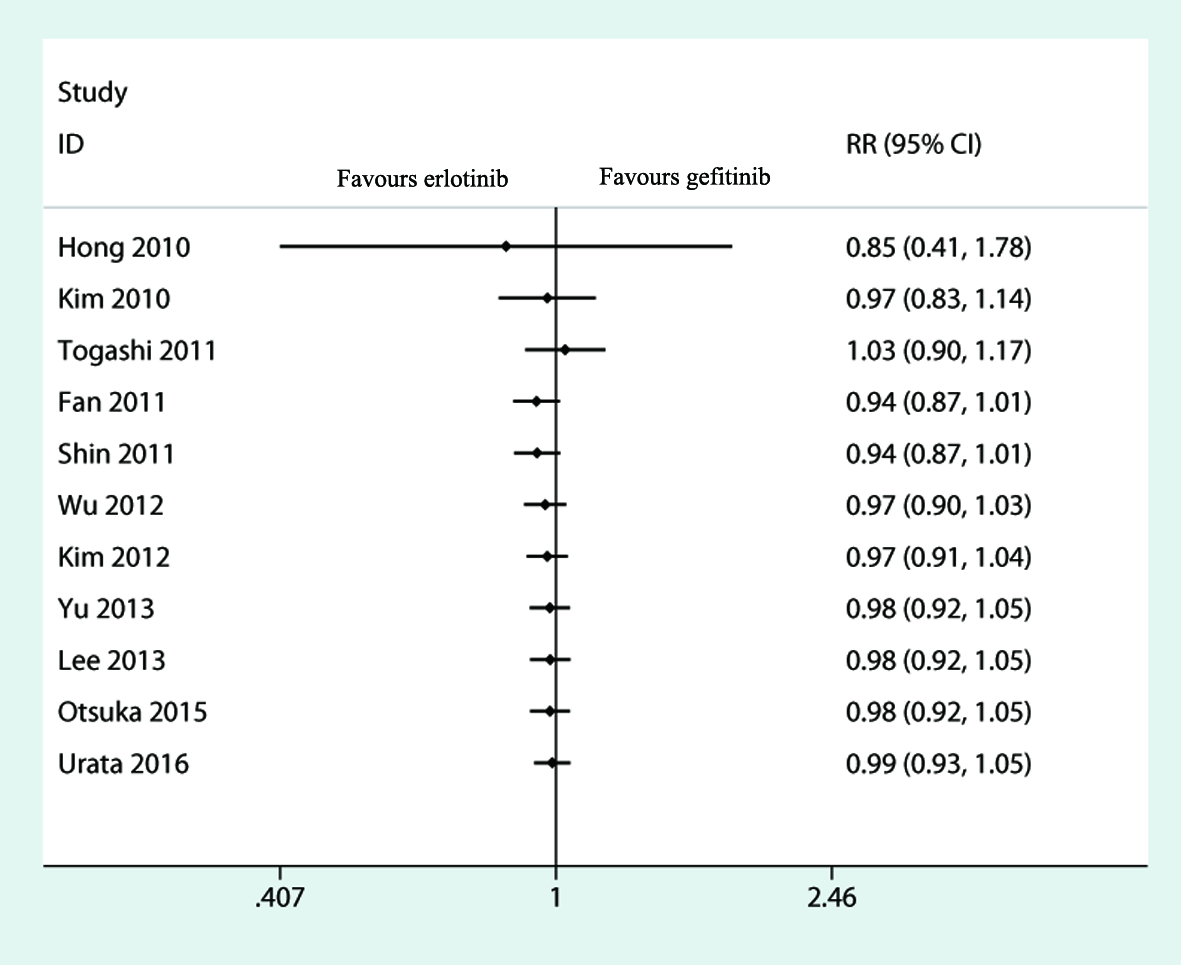

Supplement: Supplementary file 4 — Figure S4. Cumulative meta-analysis related to DCR associated with gefitinib versus erlotinib. (TIF 1379 kb) [file 12885_2018_4685_MOESM4_ESM.tif]

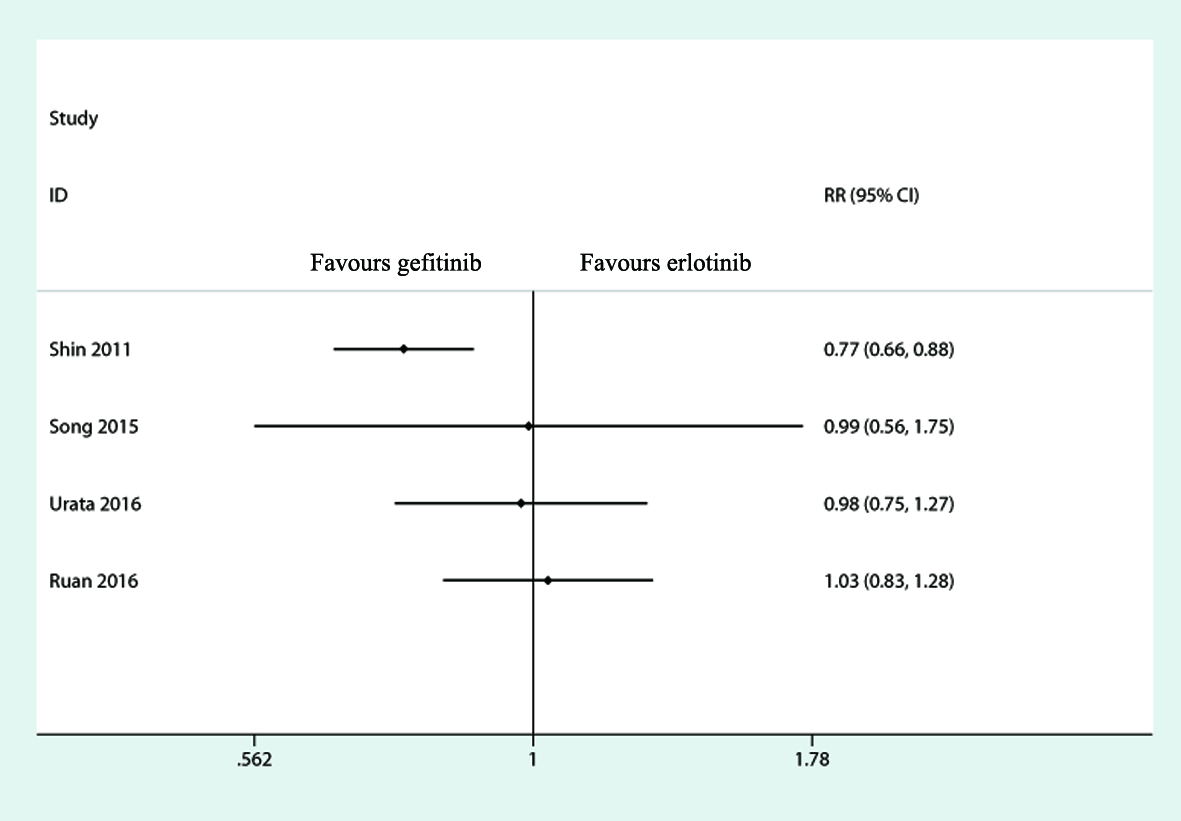

Supplement: Supplementary file 5 — Figure S5. Cumulative meta-analysis related to total AEs associated with gefitinib versus erlotinib. (TIFF 999 kb) [file 12885_2018_4685_MOESM5_ESM.tiff]

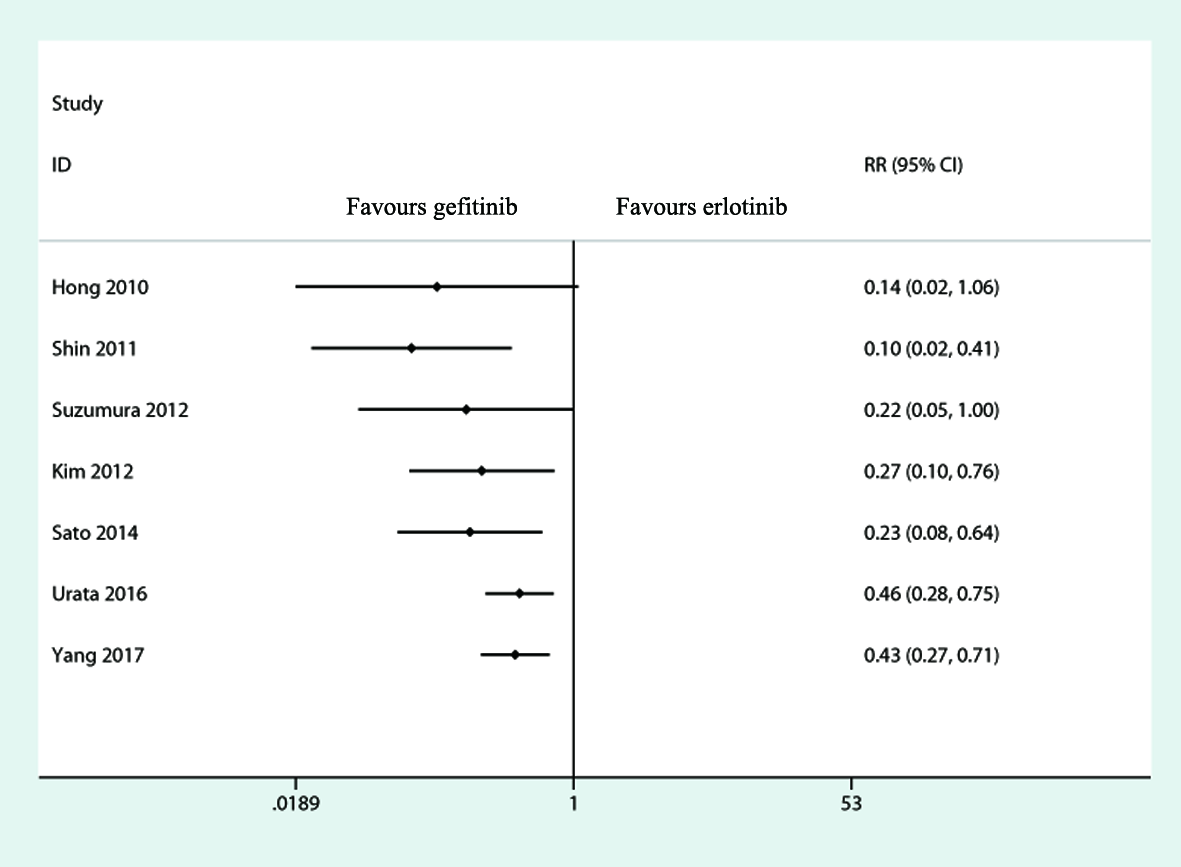

Supplement: Supplementary file 6 — Figure S6. Cumulative meta-analysis related to grade 3–5 AEs associated with gefitinib versus erlotinib. (TIFF 1104 kb) [file 12885_2018_4685_MOESM6_ESM.tiff]
